# Descriptive Analysis of Reported Adverse Events Associated with Vitiligo Medications Using FDA Adverse Event Reporting System (FAERS) Databases 2013–2023

**DOI:** 10.3390/diseases13070208

**Published:** 2025-07-02

**Authors:** Saleh F. Alqifari, Musaab Habibulla Gari, Jeff J. Guo, Shoroq Alamin, Aya K. Esmail, Abdullah K. Esmail, Heba R. Hamad, Ahmed Aljabri, Amirah M. Alatawi, Laila A. Albishi, Mohammed Olaythah Alraddadi, Helal F. Hetta

**Affiliations:** 1 Faculty of Pharmacy, University of Tabuk, Tabuk 47713, Saudi Arabia; salqifari@ut.edu.sa; 2 James L. Winkle College of Pharmacy, University of Cincinnati Academic Health Center, Cincinnati, OH 45267, USA; garimh@mail.uc.edu (M.H.G.); guoje@ucmail.uc.edu (J.J.G.); 3 College of Medicine, Sulaiman Alrajhi University, Al Bukairiyah 52726, Saudi Arabia; shoroqalamin1998@gmail.com (S.A.); ayakhaledesmail@gmail.com (A.K.E.); abdullahaghbary@gmail.com (A.K.E.); drhebarhh@gmail.com (H.R.H.); 4 Department of Pharmacy Practice, Faculty of Pharmacy, King Abdulaziz University, Jeddah 22254, Saudi Arabia; amaljabri@kau.edu.sa; 5 Department of Family and Community Medicine, Faculty of Medicine, University of Tabuk, Tabuk 47713, Saudi Arabia; am.alatawi@ut.edu.sa; 6 Pediatric Department, Faculty of Medicine, University of Tabuk, Tabuk 47713, Saudi Arabia; lalbishi@ut.edu.sa; 7 Department of Internal Medicine, Faculty of Medicine, University of Tabuk, Tabuk 47713, Saudi Arabia; malraddadi@ut.edu.sa; 8 Division of Microbiology, Immunology and Biotechnology, Department of Natural Products and Alternative Medicine, Faculty of Pharmacy, University of Tabuk, Tabuk 71491, Saudi Arabia

**Keywords:** vitiligo, adverse events, FDA adverse event reporting system (FAERS), calcineurine inhibitors, ruxolitinib

## Abstract

Vitiligo, an autoimmune disorder causing depigmented skin patches, includes two types, segmental (SV) and non-segmental (NSV). Previously, NSV was off-label treated using Calcineurine inhibitors (Tacrolimus and Pimecrolimus). In 2022, the FDA approved Ruxolitinib cream, targeting the JAK/STAT pathway for NSV treatment based on promising results. This research conducts a retrospective descriptive safety assessment of Tacrolimus, Pimecrolimus, and Ruxolitinib safety in vitiligo treatment, utilizing the FDA Adverse Event Reporting System (FAERS) database spanning the period from 2013 to 2023 and including patients aged 2 years and above, encompassing both brand and generic names. A total of 844 adverse event reports involving 388 patients were extracted and categorized into dermatological and systemic groups for analysis. Tacrolimus resulted in 12 hospitalizations, two life-threatening events, and four disabilities. Pimecrolimus exhibited urticaria and pigmentation disorders, with tooth fracture as the primary systemic event. Pericarditis was the predominant systemic side effect of Ruxolitinib, followed by anemia, headache, and urosepsis. Local dermatological side effects reported were generally mild, not warranting treatment cessation. In conclusion, vitiligo significantly impacts patients’ psychological well-being, necessitating continuous post-marketing safety monitoring for topical medications.

## 1. Introduction

Vitiligo is an autoimmune condition caused by the destruction of melanocytes, resulting in depigmented patches of skin. The prevalence of this disease ranges from 0.5 to 2% worldwide, and it can affect both genders equally at any age, with peak incidence in the second and third decades of life [1]. Clinically, it presents as depigmented macules and patches on any part of the body, which can have a major impact on the patient’s quality of life. The two main types of vitiligo recognized by a global consensus in 2011 were segmental vitiligo (SV), where lesions often appear unilaterally and asymmetrically, and non-segmental vitiligo (NSV), which is more common and results in symmetrical and bilateral white patches on the skin [1]. SV is characterized by its early onset, stable disease progression, and resistance to treatment, while NSV has a delayed onset, progressive course, and high susceptibility to treatment, which makes it the target for most newly discovered treatment agents [2].

The pathogenesis of Vitiligo is hypothesized to be a multifactorial process involving both genetic and non-genetic elements. The inheritance pattern of vitiligo is polygenic, with nearly 50 affected susceptibility loci presenting incomplete penetrance. The genetic basis of other autoimmune disorders that are epidemiologically linked to vitiligo has been shown to be closely related to the genetic basis of vitiligo [3,4]. The specific process behind the development of vitiligo remains unknown, but multiple theories have been proposed. The autoimmune hypothesis is considered a widely accepted theory. It suggests the disease is triggered and mediated by interferon-gamma (IFN-γ), an immune-derived cytokine that activates the JAK–STAT pathway to recruit CD8+ T cells, which in turn drives melanocyte destruction via detachment and apoptosis [5]. This results in the white patches on the skin that are typical of the disease. Vitiligo is considered an unpredictable disease that can go through periods of flare-ups and remissions [1]. The biochemical hypothesis suggests that melanocytes are destroyed by the build-up of reactive oxygen species (ROS) inside melanocyte cells, creating an imbalanced redox state, which leads to the development of depigmented macules [6]. The neural theory of vitiligo suggests that dysfunction within the sympathetic nervous system (SNS) might lead to depigmentation, as evident by increased cutaneous blood flow, hyperhidrosis, and emotional distress in SV lesions compared to normal skin. Additionally, multiple studies found that Stress-induced neuropeptide release, elevated nerve growth factor (NGF), and catecholamine metabolite levels may suggest an important role for the neural factors in vitiligo development [6]. Finally, the integrated (convergence) hypothesis states that vitiligo is developed by an interplay between all the proposed pathways rather than resulting from one pathophysiological mechanism alone [5,6].

The critical first step in the management of vitiligo is knowing that effective treatments are available and that it is more than a solely cosmetic condition. The main goals of vitiligo management are to slow down the disease progression, stabilize the hypopigmentation, and promote repigmentation. The European Dermatology Forum’s Vitiligo Subcommittee has developed guidelines for the diagnosis and treatment of vitiligo based on the best available evidence and expert opinions. The treatments were classified into four categories, from first- to fourth-line options. The first-line treatments are topical medications (corticosteroids and calcineurin inhibitors like Tacrolimus and Pimecrolimus). The second-line treatments are phototherapy, narrow-band ultraviolet B (NB-UVB), psoralen plus UVA (PUVA), and systemic steroid therapy. The third- and fourth-line treatments are surgical methods of skin grafting, including depigmentation therapies [7]. The aforementioned treatments are the mainstay in managing vitiligo, but they have limited success rates.

The off-label first-line topical calcineurin inhibitors (Tacrolimus and Pimecrolimus) are specifically more effective when applied on the face and neck than on the hands and feet [8]. They function through promoting the replication and migration of melanocytes and melanoblasts by multiple mechanisms, including downregulating interleukin (IL)-2 and tumor necrosis factor-α (TNF-α) production in lymphocytes, enhancing matrix metalloproteinase (MMP-2 and MMP-9) production, and playing an antioxidant role, preventing melanocyte destruction [8,9].

Recently, the U.S. Food and Drug Administration (FDA) gave its approval on 18 July 2022, for a 1.5% Ruxolitinib cream that can be applied topically on the affected skin to treat NSV. This is the first and only medication that has been approved by the FDA for repigmentation in patients aged 12 years and older [1]. The previous first-line agents, including Calcineurin inhibitors, were used off-label for vitiligo treatment [9]. Ruxolitinib is part of the family of drugs called Janus Kinase (JAK) inhibitors. It acts mainly by targeting the activation of JAK1 and JAK2, which in turn leads to inhibition in the signal transducer and activator of transcription (STAT) and blockage of the IFN-γ-mediated pathway, thus stopping the JAK/STAT pathway [10]. Ultimately, this protects melanocytes from damage and allows the potential recovery and regeneration of vitiligo-affected skin [11].

Post-marketing data regarding the adverse events and medical errors associated with the use of Tacrolimus, Pimecrolimus, Ruxolitinib, and other drugs are documented in the United States of America (USA) through a large database known as the FDA Adverse Event Reporting System (FAERS) [12]. The data are reported by the healthcare professionals (e.g., physicians, nurses, etc.) or the consumers (e.g., patients, family members, caretakers, etc.) either directly to the FDA or indirectly to the products’ manufacturers. The FAERS database is structured to follow the international safety reporting guidance issued by the International Conference on Harmonisation (ICH E2B). The Medical Dictionary for Regulatory Activities (MedDRA) is used to code adverse events and medication errors [13].

Our study aims to investigate and describe the FAERS-reported post-marketing adverse events from 2013 to 2023 associated with the topical medications used in vitiligo treatment in the USA, including Tacrolimus, Pimecrolimus, and the newly approved Ruxolitinib cream.

## 2. Materials and Methods

### 2.1. Study Design and Source Data

This study was designed as a retrospective, descriptive analysis of all reported adverse events (AEs) associated specifically with vitiligo medications, namely tacrolimus, Pimecrolimus, and Ruxolitinib. The data were extracted from the FDA Adverse Event Reporting System (FAERS), which is a database comprising information on adverse event reports, medication administration errors, and product quality complaints that resulted in adverse events and were submitted to the FDA. Its primary objective is to assist the FDA in monitoring the safety of drugs and therapeutic biologic products post-market release. Adverse events and medication errors are classified using terms from the Medical Dictionary for Regulatory Activities (MedDRA) terminology.

### 2.2. Patient Selection and Data Collection

We included patients aged 2 years and older, both genders (female and male), and both vitiligo types (SV and NSV). The data were extracted from the FDA FAERS spanning the period from 2013 to 2023. Database searches were conducted utilizing both the brand and generic names of each medication, referring to the primary adverse event associated with the drug name. The search was limited to entries where the drug was listed as the primary suspect and the indication was vitiligo. No additional filters were applied for reporter type, report source, or outcome severity. All adverse events were extracted from the column of “preferred term or PT” in the FAERS database, and we included all adverse event terms extracted from FAERS and did not exclude any terms that may not match the Medical Dictionary for Regulatory Activities (MedDRA) preferred terms.

### 2.3. Study Outcomes and Statistical Analysis:

During the study, we included all adverse event reports of the studied medications, namely tacrolimus, Pimecrolimus, and Ruxolitinib. Adverse events were categorized into dermatological and systemic groups for analysis. The total number of adverse event reports, cases, adverse reactions, and outcomes was calculated by adding the data for all years for each medication. Notably, these adverse event outcomes are not jointly exclusive, so the sum of the percentages exceeds 100 percent, as a patient can encounter more than one of these outcomes simultaneously. All analyses were conducted using Microsoft Excel 2023 Version 2312 (Microsoft Corporation, Redmond, WA, USA).

## 3. Results

In a comprehensive analysis spanning from the first quarter of 2013 to the second quarter of 2023, we examined the adverse events associated with vitiligo medications using data from the FDA Adverse Event Reporting System (FAERS). This study encompassed 844 unique adverse event reports submitted to FAERS involving a total of 388 patients. Notably, the mean age of patients was 35.41 [SD 23.77] years. The adverse events’ occurrences were nearly equally distributed between males (40.19%) and females (59.81%) in the FAERS dataset. Table 1 depicts the Adverse event reports’ pattern during the study period. The surge in submitted Ruxolitinib-related reports indicates increased usage after its approval date by the FDA.

Table 2 shows that with the three topical agents, no reported adverse events were severe enough to result in hospitalization, life-threatening condition, or disability, except in the topical calcineurin inhibitor Tacrolimus, which resulted in 12 reported hospitalizations, two life-threatening adverse events, and four disabilities.

For topical tacrolimus, Table 3 shows the dermatological adverse events associated with the use of the medication. The total reported dermatological adverse events were 59, the most common being burning sensation, application site pain, and application site pruritus, followed by dermatitis, dermatitis bullous, and lentigo. Table 4 illustrates a total of 90 systemic adverse events involving other systems. Aplastic anemia and haemorrhage were the most commonly reported systemic adverse events, with four reports for each.

For topical Pimecrolimus, Table 3 shows urticaria and pigmentation disorders as the most commonly reported dermatological adverse events, followed by application site pain, pruritus, skin burning sensation, and skin exfoliation. On the other hand, Table 4 shows tooth fracture as the most common systemic adverse event, followed by spontaneous abortion, COVID-19, dyspnoea, GI disorders, headache, influenza, lung disorders, malaise, viral pneumonia, rhinitis, and viral infection.

For topical Ruxolitinib, Table 3 shows that the most commonly reported dermatological event is acne, followed by application site pain, pruritus, rash, skin abrasion, and skin hyperpigmentation. On the other hand, Table 4 shows that the most commonly reported systemic side effect is pericarditis, followed by anemia, headache, and urosepsis.

## 4. Discussion

A retrospective descriptive study was conducted to analyze the reported post-marketing adverse events associated with vitiligo medications from 2013 through 2023, utilizing data from the FAERS database in the USA. This study aims to provide a comprehensive overview of the most common adverse events associated with vitiligo medications to ensure the safety of these agents in their post-marketing phase and to investigate the probable emergence of new adverse events.

The dataset results showed an expected low to negligible rate of hospitalization, life-threatening conditions, and disability following the use of topical Ruxolitinib, Pimecrolimus, and Tacrolimus. However, cases of hospitalization, life-threatening adverse events, and disabilities were reported with tacrolimus alone, accounting for 12 hospitalizations, 2 life-threatening events, and 4 cases of disability. This is anticipated as topical medications are less likely to reach the systemic circulation, maintaining a subtherapeutic plasma level and thus reducing the rate of serious systemic adverse events [14,15]. A study has shown that systemic exposure to topical Tacrolimus was detected in 39% of the blood samples, while topical Pimecrolimus was detected in 12% only. Additionally, among these samples with detectable systemic exposure, the blood concentration of Tacrolimus was higher than Pimecrolimus [15]. This might explain the occurrence of serious systemic adverse events, although very rare, with topical Tacrolimus alone in the post-marketing data. Although the database illustrated some serious side effects that necessitate hospital admission in the other two medications, such as pericarditis in patients who were given Ruxolitinib, these cases might have been reported by a non-hospital facility, where reporting hospitalization could be ignored.

Tacrolimus and Pimecrolimus are immunomodulatory drugs that inhibit calcineurin, a crucial enzyme in T-cell activation. By suppressing T-cell function, these medications exert anti-inflammatory effects, making them valuable in treating inflammatory skin conditions. This mechanism provides insight into why a substantial number of observed dermatological adverse events are likely attributed to the local immunomodulatory effects of these drugs, particularly with calcineurin inhibitors.

The most commonly reported post-marketing dermatological adverse events identified in our study for the topical calcineurin inhibitor Tacrolimus were nine burning sensation reports, six application site pain reports, six application site pruritus reports, and four erythema reports. Likewise, Pimecrolimus’s most commonly reported adverse events were three pigmentation disorder reports, three urticaria reports, two burning sensation reports, and two application site pain and pruritus reports. This was consistent with a meta-analysis analyzing the treatment outcome of topical calcineurin inhibitors in vitiligo treatment, which identified a burning sensation (9.8%), pruritus (7.4%), and erythema (2.4%) as the most common adverse events among 296 treated vitiligo patients [8]. These side effects were temporary and did not lead to therapy discontinuation or additional treatment [8]. These findings align with a previously published article, which highlighted that tacrolimus use may lead to mild-to-moderate burning sensations, erythema, and pruritus [16]. The article emphasized the occurrence of various dermatological effects, including folliculitis, acne, Kaposi’s varicelliform eruptions, eczema herpeticum, and herpes simplex infections [16]. Other dermatological adverse event reports from the literature included heightened skin sensitivity to temperature extremes and alcohol intolerance with topical Tacrolimus use in atopic dermatitis, along with documenting uncommon side effects such as recurrent skin tags, rosacea-like granulomatous eruptions, rosaceiform dermatitis, mucosal hyperpigmentation, tinea incognito, molluscum contagiosum, and verruca vulgaris [16]. Likewise, Pimecrolimus was noted for primarily inducing a burning sensation on the skin, with minimal cases requiring discontinuation [17].

Additionally, topical Tacrolimus use in our data was noted to be associated with the occurrence of serious systemic adverse events; the most commonly reported were four aplastic anemia events, four hemorrhage events, and three QT prolongation events. Literature documented that therapy with immunosuppressive medications like oral Tacrolimus could rarely result in anemia due to bone marrow suppression [18]. Although Tacrolimus is thought to have a minimal likelihood of causing myelosuppression, therapy with Tacrolimus was found to be closely associated with pure red cell aplasia (PRCA) [19]. Surprisingly, in regard to the QTc prolongation, Tacrolimus in the oral formulation, which has a higher probability of causing systemic side effects than the topical formulation, was suggested to be the primary therapeutic option for patients who suffer from prolonged QT interval during their immunosuppressive treatment course [20]. On the other hand, several case report studies documented the occurrence of prolonged QT intervals after the use of Tacrolimus [21]. However, these cases are rare, and there is not enough evidence to support the association.

Ruxolitinib is available in two formulations, both oral and topical. The topical formula was approved by the FDA for use in vitiligo patients. Regarding its safety profile, since the topical formula results in a lower serum concentration of Ruxolitinib, it is expected that the adverse events will be mostly local [22]. This was also evident by the results of our analysis in which the reported adverse events were mostly dermatological, including acne, pruritis, rash, skin abrasion, and hyperpigmentation as well as other application site adverse effects such as pain, erythema, irritation, etc… These findings are in line with the most commonly adverse events reported in the TRuE-V1 and TRuE-V2 double-blinded trials; namely application site acne and pruritis (5.8%, 5.1% respectively) [23]. However, two other trials assessing the safety and efficacy of Ruxolitinib in atopic dermatitis patients have reported nasopharyngitis and upper respiratory tract infections as the most common presenting adverse events, with application site pruritis and pain being less frequent [24,25]. On the other hand, oral formula has been reported to be associated with systemic side effects, mainly myelosuppression resulting in cytopenia, which may lead to anemia, thrombocytopenia, leukopenia, and neutropenia [26]. Infections, namely viral infections, tuberculosis, and fungal infections [26]. However, since topical formulas usually lead to a local response rather than a systemic one [22], the side effect profile of Ruxolitinib cream has been reported by trials to be less severe and more tolerable [22,23,27]. Nonetheless, our analysis showed several reported systemic adverse events such as anaemia, pericarditis, and urosepsis.

In addition to the anticipated dermatological effects, our study revealed other systemic adverse events not typically linked to topical treatment. For example, pericarditis and urosepsis reported with Ruxolitinib, as well as aplastic anaemia and QT prolongation with Tacrolimus, indicate safety concerns that necessitate further investigation. Despite the minimised systemic absorption achieved through topical administration, these events suggest either rare systemic exposure, or other patient-specific characteristics and comorbidity not acknowledged through FAERS, like compromised skin barriers.

### Study Limitations

This study has several limitations. First, due to the low number of adverse event reports for some medications, statistical comparisons such as odds ratios or other standardized risk measures could not be performed. Therefore, we relied solely on descriptive frequency data. Second, the FAERS database is based on voluntary, self-reported cases, which introduces inherent reporting bias. We did not apply any statistical correction for this bias, and our results should be interpreted with this limitation in mind. Third, the database does not include detailed clinical information such as treatment dosage, duration, comorbidities, or total patient exposure. As such, it is not possible to estimate the true incidence of adverse events or determine whether these events were caused by the medication or by other underlying factors. Consequently, comparisons across medications are based on absolute report counts rather than per-patient risk. Despite these limitations, this study provides useful insight into the types and frequencies of post-marketing adverse events associated with topical treatments for vitiligo.

## 5. Conclusions

Vitiligo is an important dermatological disease that has a significant effect on the patient’s psychological quality of life. The first line topical calcineurin inhibitors, Tacrolimus and Pimecrolimus, were used off-label in non-segmental vitiligo for a long time before the official approval of topical tacrolimus. Post-marketing safety monitoring of these topical medications remains essential. In our analysis, Tacrolimus was observed to be associated with serious systemic adverse events leading to hospitalization, life-threatening conditions, and disabilities. Although Pimecrolimus and Ruxolitinib did not show adverse event outcomes of similar severity, critical systemic events such as pericarditis, anaemia, and urosepsis were reported with Ruxolitinib, while spontaneous abortion and viral infections were linked to Pimecrolimus. These results indicate a comparatively advantageous, yet not free of risk, systemic safety profile for Pimecrolimus and Ruxolitinib in contrast to Tacrolimus.

## Figures and Tables

**Table 1:** Number of adverse event reports submitted following the usage in vitiligo patients.

	Pimecrolimus	Tacrolimus	Ruxolitinib
2013	15	52	N/A
2014	6	45	N/A
2015	0	38	N/A
2016	1	15	N/A
2017	18	15	N/A
2018	2	23	N/A
2019	15	13	N/A
2020	2	15	N/A
2021	14	44	2
2022	25	60	3
2023	1	21	399

N/A “ Not applicable”.

**Table 2:** Type of adverse event outcomes associated with vitiligo medications.

Drug Name	Total Adverse Events	Hospitalization	Life-Threatening	Disability	Other
Tacrolimus	341	12	2	4	149
Pimecrolimus	97	0	0	0	21
Ruxolitinib	406	0	0	0	10

**Table 3:** The Dermatological Adverse Events Frequencies for Tacrolimus, Pimecrolimus and Ruxolitinib.

Adverse Event	Tacrolimus	Pimecrolimus	Ruxolitinib
Acne	2	0	6
Application site acne	0	0	3
Application site discoloration	2	0	2
Application site erythema	2	0	1
Application site irritability	0	0	1
Application site irritation	0	0	1
Application site pain	6	2	5
Application site paraesthesia	0	0	1
Application site pruritus	0	0	2
Application site reaction	0	0	1
Application site ulcer	0	0	1
Basal cell carcinoma	1	0	0
Blister	4	0	2
Chloasma	0	0	2
Condition aggravated	0	0	3
Dermatitis	1	0	2
Dermatitis bullous	4	0	0
Drug hypersensitivity	0	0	3
Dry skin	0	0	2
Erythema	4	0	3
Eyelash discolouration	0	0	1
Flushing	1	0	0
Folliculitis	0	0	3
Hair growth abnormal	1	0	0
Herpes zoster	1	0	2
Hypersensitivity	0	0	2
Hypertrichosis	1	0	0
Lentigo	3	0	0
Onycholysis	0	0	1
Pain of the skin	0	2	2
Pigmentation disorder	0	3	0
Porokeratosis	1	0	0
Pruritus	2	2	5
Rash	2	1	5
Rash papule	0	0	1
Skin abrasion	2	0	5
Skin burning sensation	9	2	1
Skin discolouration	0	1	2
Skin disorder	0	0	1
Skin exfoliation	0	2	3
Skin haemorrhage	0	0	1
Skin hyperpigmentation	1	0	5
Skin injury	0	0	1
Skin irritation	0	0	2
Skin tightness	0	0	1
Solar lentigo	0	0	2
Squamous cell carcinoma	2	0	0
Urticaria	1	3	1
Yellow skin	0	0	1
**Total**	59	18	89

**Table 4:** The Systemic Adverse Events Frequencies for Tacrolimus, Pimecrolimus and Ruxolitinib.

Adverse Event	Tacrolimus	Pimecrolimus	Ruxolitinib
Abdominal pain upper	0	0	1
Abortion spontaneous	2	2	0
Alcohol intolerance	1	0	0
Anaemia	0	0	5
Anaphylactic reaction	1	0	0
Anosmia	1	0	0
Antinuclear antibody positive	1	0	1
Aplastic anaemia	4	0	0
Arthralgia	1	1	0
Asthenopia	1	0	0
Blindness unilateral	0	0	1
Breath odour	1	0	0
Cataract	0	0	2
Cerebrovascular accident	2	0	0
Cheilitis	1	0	0
Chills	0	0	2
Colitis ulcerative	2	0	0
Conjunctival haemorrhage	2	0	0
Corneal irritation	1	0	0
Cough	1	0	1
COVID-19	0	2	1
Deep vein thrombosis	0	0	1
Dehydration	0	0	2
Diarrhoea	1	0	0
Discomfort	1	0	1
Diverticulitis	0	0	1
Dizziness	0	0	1
Drug hypersensitivity	3	0	0
Drug intolerance	0	0	1
Drug-induced liver injury	1	0	0
Dry mouth	1	0	1
Dyspnoea	0	2	0
Dysstasia	1	0	1
Dysuria	1	0	1
Effusion	2	0	0
Electrocardiogram QT prolonged	3	0	0
Eosinophil counts increased	1	0	1
Epilepsy	1	0	0
Eye irritation	1	0	0
Facial paralysis	1	0	0
Fall	0	0	1
Feeling abnormal	0	1	0
Feeling cold	0	0	1
Gastrointestinal disorder	0	2	0
Gastrointestinal infection	0	1	0
Globulins increased	1	0	1
Grip strength decreased	1	0	0
Haematuria	1	0	1
Haemorrhage	4	0	0
Headache	1	2	4
Heavy menstrual bleeding	2	0	0
Hepatitis acute	1	0	0
Hip arthroplasty	1	0	0
Hordeolum	0	0	1
Hypertension	1	0	2
Hyperthyroidism	2	0	0
Hypogammaglobulinemia	1	0	0
Ill-defined disorder	0	0	3
Impaired quality of life	1	0	0
Infection	0	0	1
Infection reactivation	1	0	0
Influenza	0	2	0
Influenza-like illness	1	0	1
Insomnia	0	0	2
Iridocyclitis	1	0	0
Lip blister	0	0	1
Liver disorder	0	0	2
Liver injury	1	0	0
Lung disorder	0	2	0
Lymphadenopathy	1	0	0
Malaise	0	2	0
Maternal exposure during pregnancy	3	2	1
Mood swings	0	0	1
Muscle disorder	0	1	0
Muscle spasms	0	0	1
Myalgia	1	0	1
Myelosuppression	2	0	0
Nasal congestion	1	0	0
Nasopharyngitis	1	0	1
Nausea	0	0	1
Neoplasm malignant	2	0	0
Oropharyngeal pain	0	0	2
Orthostatic hypertension	0	0	2
Orthostatic hypotension	0	0	1
Pain	0	0	1
Pain in the extremity	2	0	1
Palpitations	0	0	1
Penis disorder	1	0	0
Pericarditis	0	0	6
Pharyngeal swelling	0	0	1
Pharyngitis	1	0	1
Pneumonia viral	0	2	0
Prostate cancer	0	0	1
Pulmonary embolism	0	0	1
Quality of life decreased	0	0	1
Rhinitis	0	2	0
Rhinorrhoea	0	0	1
SARS-CoV-2 test positive	0	0	1
Sjogren’s syndrome	1	0	1
Spinal pain	1	0	0
Stillbirth	1	0	0
Sweat gland tumour	0	0	2
T-cell lymphoma	1	0	0
Throat irritation	0	0	3
Throat irritation	1	0	3
Thyroid disorder	0	0	1
Toe operation	1	0	0
Tooth loss	0	0	1
Toothache	0	0	1
Trichomegaly	2	0	0
Type 1 diabetes mellitus	0	0	1
Umbilical hernia	1	0	0
Upper limb fracture	0	0	2
Urinary tract infection	1	0	0
Urine odour abnormal	1	0	0
Urosepsis	0	0	4
Viral infection	0	2	0
Vitamin B12 deficiency	1	0	1
Vitamin D deficiency	1	0	1
Vitreous detachment	1	0	0
Weight decreased	0	1	0
White blood cell counts decreased	0	0	1
**Total**	90	32	92

## Data Availability

Not applicable.
